# Exploring COVID-19 vaccine hesitancy among young adults in Australia. A qualitative study

**DOI:** 10.1016/j.jvacx.2024.100515

**Published:** 2024-06-22

**Authors:** Zachary Sum, Ernesta Sofija, Bernadette Sebar

**Affiliations:** School of Medicine and Dentistry, Gold Coast Campus, Griffith University, 1 Parklands Dr, Southport, QLD 4222, Australia

## Abstract

**Background:**

COVID-19 vaccine hesitancy among young adults in Australia is still poorly understood. Young adults aged 25–34 years have been identified as a population subgroup where COVID-19 vaccine-hesitant views are highly prevalent.

**Objective:**

Exploring the attitudes, thoughts, feelings and social environments affecting the decision to have or not have the vaccine can provide vital transferrable lessons in future health campaigns.

**Methods:**

A qualitative phenomenological study was conducted using semi-structured phone interviews between June 2021 and July 2021. Interview questions were adopted from the World Health Organization’s guidance document ‘Data for Action: Achieving High Uptake of COVID-19 Vaccines’. Participants aged 25–34 years (n = 26) were recruited via purposive and snowball sampling methods. Data were analysed thematically.

**Results:**

Overall, participants presented themselves as highly vaccine literate, understanding their social contract with society. Many participants also did not display traditional vaccine-hesitant views. Six themes specifically regarding COVID-19 vaccine hesitancy were identified, namely (1) decision-making complexities, (2) perceived risk of COVID-19 infection, (3) media’s misrepresentation of COVID-19, (4) vaccine-related issues, (5) inconsistent government messaging and program execution, and (6) social benefits assessment. In addition, motivators to COVID-19 vaccine uptake were classified into three categories: (1) tangible benefits, (2) protecting others, and (3) mandates and incentives. Findings suggest the motivators for COVID-19 vaccine uptake in young adults depend on individual benefits, highlighting the importance of recognising and addressing personal concerns.

**Conclusion:**

There is a need to re-examine and redefine the meaning of vaccine hesitancy in young Australian adults. We offer an understanding of prospective challenges with vaccine hesitancy and potential solutions to address them. These include carefully tailored approaches regarding ongoing vaccine safety concerns and the expected personal benefits following vaccination. Combining these factors can aid in developing new methods of public engagement in the next public health crisis.

## Introduction

As of 1 January 2022, COVID-19 has infected more than 280 million people and has resulted in more than 5 million deaths globally [1]. A high vaccination rate is critical to reducing COVID-19-related morbidity and mortality and enabling the resumption of economic activity. However, as the COVID-19 vaccines were developed to market quickly, vaccine hesitancy presents a major challenge to public health teams globally and in Australia, especially during the vaccination campaign’s initial stages [2], [3].

Sentiments of vaccine hesitancy, such as unknown safety and efficacy and profiteering from medical professionals, have historically been an obstacle since the creation of the smallpox vaccine by Edward Jenner in 1798 [4], [5], [6]. Vaccine hesitancy is complex and context-specific, varying across time, place and vaccine types. Such hesitancy is heavily influenced by complacency, convenience and confidence [7]. In January 2019, general vaccine hesitancy was identified by the World Health Organization (WHO) as one of the top ten threats to public health worldwide [8].

Vaccine hesitancy is a behaviour resulting from attitudes and beliefs that influence the decision-making process, leading to either acceptance or refusal of some or all vaccines according to the recommended immunisation schedule [9]. Given the multifaceted and context-specific nature of vaccine hesitancy, WHO's Strategic Advisory Group of Experts (SAGE) has identified numerous factors that influence vaccination decision-making [10]. As a result, two models were proposed: an easy-to-understand 5C model and a more comprehensive model called The Working Group Determinants of Vaccine Hesitancy Matrix [11]. These models aimed to assess potential usefulness and applicability in informing the development of vaccine hesitancy indicators, survey questions, and interventions [10]. Most recently, the WHO’s SAGE has developed innovative tools and indicators to assess various factors influencing vaccine uptake and address vaccine hesitancy. Specifically, the Behavioural and Social Drivers (BeSD) tool has been created to evaluate the effects of behaviour and social determinants on childhood vaccinations and more recently, COVID-19 vaccination [12]. The aim is to provide decision-makers in immunisation policy, immunisation program managers, and partners with the means to comprehend and address the root causes of low vaccine uptake, in order to effectively combat under-vaccination [12].

### Perceived risk of COVID-19 infection

Globally, COVID-19 has infected adults of all ages [13]. Since the first reported cases of COVID-19 in January 2020, 43.5 % of people infected with COVID-19 in Australia have been adults aged between 20 and 39 years [14]. This age group makes up many essential workers in retail, food processing, warehousing and healthcare services, where occupational and behavioural factors can reduce social distancing and relaxed mask-wearing [15], [16]. Accumulating evidence indicates a strong age-related gradient to long-term morbidity and death after contracting COVID-19 [17]. Younger adults formed the bulk of those infected around the world and been identified as the primary transmitters of the virus in the community, with older populations and those with medical vulnerabilities being most susceptible to severe illness and mortality [18], [19]. Only a small number of deaths were reported among young adults in high-income countries [20]. As a result of lower health consequences post-COVID-19 infection among young adults, it is demonstrated that an inverse relationship between COVID-19 vaccine hesitancy and one’s perceived risk of a severe illness from COVID-19 [21]. Those who perceive themselves as being able to recover from COVID-19 had higher hesitancy scores as compared to those who perceive themselves as at a high risk of severe COVID-19 illness [21], mirroring trends reported in France and Malaysia [22], [23].

COVID-19 vaccine hesitancy research in 2020 was predominantly quantitative and conducted before the availability of licensed COVID-19 vaccines [24], [25]. Australia’s COVID-19 mass vaccination program started in February 2021, and vaccine hesitancy levels are anticipated to differ greatly from previously published cross-sectional studies [26], [27], [28]. In Australia, a longitudinal study conducted between August 2020 and January 2021 observed a general decline in willingness to vaccinate against COVID-19 [25]. Notably, the study identified three primary factors contributing to this decline: individuals who believed that the COVID-19 pandemic was being exaggerated, those with low confidence in hospitals and the healthcare system, and those with pessimistic views about the upcoming year [25].

A survey conducted in Australia demonstrated COVID-19 vaccine hesitancy to be between 14.4 % and 18.7 % of the eligible population [25]. The authors found that young people aged 25 to 34 years were the most hesitant (28.8 %) as compared to their younger and older counterparts (18–24 years old, 15.5 %, 35–44 years old, 24.7 %; 55–64 years old, 19.3 %; and 65–74 years old, 12.1 %) [25]. Similarly, another survey revealed that 35 % of younger Australians aged 25 to 34 years were more likely to have concerns about COVID-19 vaccine safety than those 50–54 years old (32 %), 55–64 years old (30 %), and over 75 years old (10 %) [29].

### Research gaps and aims

To achieve high vaccination coverage, it is essential to encourage vaccination among the least vulnerable population, notably healthy young adults. While quantitative research indicates that young adults show greater hesitancy towards COVID-19 vaccination [21], [25], [29], [30], [31], [32], [33], [34], [35], limited qualitative research has been conducted to explore this trend in depth [36], [37], [38]. Furthermore, young adults in Australia have demonstrated contrasting levels of COVID-19 vaccine willingness, with research stating those aged 18–24 years are the least hesitant while those aged 25–34 years are the most hesitant [25]. Therefore, this evidence provides a unique opportunity to address the challenges faced by this particularly hesitant group of young adults in Australia. Although COVID-19 vaccinations are not mandatory for all young individuals in Australia, researching COVID-19 vaccine hesitancy within this demographic could offer valuable insights into the perception of new-to-market vaccine adoption. Such insights could provide crucial guidance to key health organisations to enhance future vaccination initiatives and promote increased vaccine uptake. For this reason, this qualitative study was conducted to investigate and characterise vaccine hesitancy among Australia’s most COVID-19 vaccine-hesitant population − those aged 25–34 years.

## Methods

### Study setting and timing

The data collection was conducted from 15th June to 21st July 2021 through telephone interviews with participants residing in Australia. It is important to note that different states and territories experienced varied shifts of COVID-19 infections during the data collection period and imposed varying levels of lockdown restrictions and other measures. As of June 2021, adults aged 25 to 34 who were key essential workers or immunocompromised were eligible for a COVID-19 vaccine. A notable increase in vaccine hesitancy was observed following reports of adverse reactions, including rare blood clots associated with the AstraZeneca Vaxzevria COVID-19 vaccine. Consequently, during that period, only a marginal proportion (2.1 %) of the eligible Australian adult population had received both doses of a COVID-19 vaccine [39].

### Study population and sample size

The study population consisted of COVID-19 vaccine-hesitant young adults aged between 25 to 34 years. The study’s inclusion criteria included those who 1) did not require a COVID-19 vaccination for work or study, 2) had not been vaccinated with a COVID-19 vaccine, and 3) had not been diagnosed with COVID-19 at the time of the interview or previously. The study sample consisted of 26 participants.

### Data collection

A descriptive phenomenological approach uncovered the study participants’ attitudes, thoughts and feelings around COVID-19 vaccine uptake.

Semi-structured interviews were conducted using a predetermined set of questions based on the Behavioural and Social Drivers (BeSD) of the COVID-19 vaccination framework from the WHO SAGE document *Data for action: Achieving high uptake of COVID-19 vaccines* [40]. The BeSD framework identified 'thinking and feeling' and 'social processes' as two domains closely linked with vaccine hesitancy, or motivational conflict, and 'practical issues' as a critical domain moderating the relationship between vaccine hesitancy and vaccine uptake. 'Thinking and feeling' was operationalised through questions assessing information sources, rumours, sentiments about COVID-19 vaccines, and strategies to enhance vaccine confidence. 'Social processes' were operationalised by evaluating individual and perceived community attitudes toward COVID-19 vaccines. 'Practical issues' were assessed through questions examining the impact of the COVID-19 pandemic on health and daily activities, along with barriers and facilitators to COVID-19 vaccination within the community. Minor modifications were made to the sequence of interview questions (asking ‘practical issues’ questions at the start instead of at the end of the interview), and the revised qualitative interview guide was used throughout the whole study period. While not explicitly developed to evaluate vaccine hesitancy, this framework was utilised to gain insight into vaccine hesitancy from a perspective that contrasts with the factors driving vaccination.

Participants were recruited by convenience sampling using purposive and snowball methods via social media advertisements and the University’s broadcast mail. This method was suitable because of the high usage of technology and social media in the target age group. Convenience sampling was also performed at the author’s (ZS) workplace, where the author was involved in implementing a COVID-19 vaccination program, thus allowing access to both potential male and female participants.

All interviews were conducted via verbal phone conversations. Consent was obtained from all participants before the interviews. Interviews were audio-recorded and transcribed verbatim for analysis, with identifying information removed. Participants’ names were pseudonymised. Before each interview, participants’ basic demographic data were collected. The interviews lasted between 30 min and one hour, with an average mean time of 47 min.

### Data analysis

Inductive thematic analysis was used to analyse the data using NVivo by QSR International. Braun and Clarke [41] six-step framework was used to identify recurring patterns in the interview. Each transcript was read repeatedly to search for meanings and patterns. During this process, important text was identified, coded and indexed. Codes were then categorised into subthemes and themes to form a coherent pattern and relationship structure. The themes identified were classified into two main categories: 1) factors of COVID-19 vaccine hesitancy and 2) potential motivating factors to COVID-19 vaccine uptake. This process was repeated until data saturation was established. Throughout the analysis, co-authors (ES and BS) examined, reviewed and finalised the themes for relevance, accuracy and robustness.

## Research team and Reflexivity

The first author, ZS, was a pharmacist immuniser in Australia’s COVID-19 vaccination program. Any preconceived notions and assumptions about vaccine hesitancy were regularly discussed with co-authors ES and BS throughout data collection and analysis to guard against and reduce bias. A detailed audit trail documenting the decision-making process used during data analysis was maintained. To minimise bias, a second round of coding was performed to ensure that alternative interpretations of the data were considered.

Ethics approval for this research was obtained from the Griffith University Human Research Ethics Committee (GU Reference Number: 2021/401).

## Results

### Participants

Table 1 presents the key demographic characteristics of study participants. The mean age was 28.9 years. The majority of participants were female (n = 21), born in Australia (n = 20), resided in the state of Victoria (n = 14), followed by Queensland (n = 10) and New South Wales (n = 2). One participant described themselves as a person living with a physical disability.

**Table 1:** Demographic characteristics of participants (n = 26).

Pseudonym	Age	Sex^a^	State^b^	Country of Birth	Dependent Children	Highest Education Received	Relationship Status	Housing Status	Employment
Adele	33	F	VIC	Australia	1	Certificate	Single	Renting	Part time
Brea	33	F	VIC	Australia	1	Bachelor’s degree	De facto	Living with family	Unemployed
Capri	33	F	VIC	Australia	5	High school	Married	Renting	Self-employed
Demi	29	F	VIC	Australia	1	Bachelor’s degree	Married	Home owner	Full time
Evie	26	F	VIC	Australia	0	Bachelor’s degree	Single	Living with family	Part time
Flora	25	F	VIC	Australia	0	High school	De facto	Living with family	Self-employed
Gemma	26	F	QLD	Overseas	0	Certificate	De facto	Home owner	Student
Hanna	31	F	QLD	Australia	0	Bachelor’s degree	De facto	Renting	Full time
Ivan	26	M	NSW	Australia	0	Certificate	Single	Renting	Student
Jayla	31	F	VIC	Overseas	0	Bachelor’s degree	Married	Renting	Full time
Kevin	26	M	VIC	Overseas	0	Postgraduate degree	Single	Renting	Full time
Laila	25	F	NSW	Australia	0	Diploma	De facto	Renting	Full time
Maia	31	F	QLD	Overseas	0	Postgraduate degree	De facto	Renting	Student
Nalani	26	F	QLD	Australia	0	Bachelor’s degree	Married	Home owner	Full time
Olive	27	F	QLD	Australia	1	Bachelor’s degree	Divorced	Renting	Student
Priya	27	F	QLD	Australia	0	Bachelor’s degree	De facto	Renting	Part time
Quinn	26	M	QLD	Australia	0	Bachelor’s degree	Single	Renting	Student
Raya	29	F	QLD	Australia	0	Certificate	De facto	Home owner	Full time
Sadie	29	F	QLD	Australia	0	Bachelor’s degree	De facto	Renting	Student
Trent	30	M	QLD	Australia	0	Postgraduate degree	Single	Renting	Part time
Una	28	F	VIC	Australia	1	Postgraduate degree	Married	Home owner	Full time
Varun	34	M	VIC	Overseas	0	Postgraduate degree	Married	Renting	Part time
Willa	30	F	VIC	Overseas	0	Bachelor’s degree	Married	Renting	Full time
Xena	27	F	VIC	Australia	0	Bachelor’s degree	De facto	Renting	Unemployed
Yasmin	32	F	VIC	Australia	2	Diploma	Married	Renting	Home duties
Zara	33	F	VIC	Australia	3	Bachelor’s degree	Married	Home owner	Full-time

*Note.*^a^: M = Male; F = Female. ^b^: QLD = Queensland; VIC = Victoria; NSW = New South Wales.

The findings presented in this section will be prefaced by describing the participants' prior experiences with vaccination. Most participants were highly supportive of vaccinations and their associated benefits to society and public health. Ivan viewed vaccinations as “really beneficial for humanity”. Participants also supported receiving necessary vaccines for employment or education purposes. Most participants also kept up to date with vaccinations during routine medical care or when required for travelling. This positive attitude to vaccinations also extended to their child/ren who received all necessary childhood vaccines under the National Immunisation Program. As one participant succinctly described it:

*“I’m vaccinated, my son is vaccinated… he is due for his six months [scheduled vaccination] next week as well, so he’s going. I haven’t had any issues. So, I’m actually really comfortable with it.” (Una)*.

Overall, the responses from participants regarding vaccinations were generally positive, with very little questioning the need, efficacy and safety of current vaccinations. This background sets the context in exploring why normally vaccine-accepting young adults are hesitant about receiving the COVID-19 vaccine. The themes identified reveal the essence of the thoughts and feelings of these vaccine-hesitant young adults and how their social behaviours influenced their decision towards a COVID-19 vaccination. The findings are presented in two sections: 1) factors contributing to COVID-19 vaccine hesitancy and 2) motivators to COVID-19 vaccine uptake.

### Factors contributing to Covid-19 vaccine hesitancy

#### Theme 1: Decision-making complexities

The decision to be vaccinated against COVID-19 was revealed as a challenging experience filled with emotions and fear. Participants stated that the overload of information made deciding whether to vaccinate particularly difficult. Based on observations of friends and families who had the COVID-19 vaccine, participant Adele expressed the lack of encouraging post-vaccination experiences by describing friends who received the vaccine as ‘not overly happy’. At the same time, Priya mentioned the overall ‘negative rhetoric around the [COVID-19] vaccine’ had dampened her vaccine confidence.

Participants also criticised the difficulty in finding reputable sources of COVID-19 vaccine information, with some describing receiving an avalanche of confusing information. Sadie explained that it was ‘not that information can’t be obtained’ but that she felt so ‘bombarded’ with information that ‘it bothers [her]’.

Conversely, some participants remarked that considering the state of the pandemic, information should be easy to locate. Instead, participants felt that other pandemic-related issues often dominated vaccine-related information participants were looking for:

*“Even if you go on government websites… there’s still nothing. It’s always about the restrictions… ‘Where is the vaccine research point, can you tell us?’. It’s not even coming into light anymore.” (Jayla)*.

Many participants had hoped for a balanced view on the COVID-19 vaccines but felt that current vaccine information was too controlled and one-sided. Varun acknowledges that he is not looking for someone to support the notion of an ‘absolutely safe vaccine’ but would prefer the government to ‘have a greater discussion’ and acknowledge the multiple perspectives of the vaccines with the public.

While presented with information about the benefits of receiving a COVID-19 vaccine, one participant conveyed her contradictory decision-making concerning the risk of COVID-19 vaccine side effects compared to her current risky health behaviours:

*“In reality, there’s a lot of things that I do in my life that are not very healthy. I drink alcohol pretty regularly. I occasionally smoke*… *But then this one thing [COVID-19 vaccine] … I’m like ‘Oh, I’ve got to be worried about that!’” (Hana)*.

Several participants also possessed contradictory requirements for themselves and society. Kevin provided a detailed thought process when it came to his decision-making dilemma:

*“When I do self-reflection about these questions… I reckon maybe I shouldn’t be selfish because the public consists of every individual. There’s no society without individuals. So as an individual, I should consider myself as a part of the society. I need to take the step to encourage others. Sometimes I think in this way, but sometimes I [am] like… ‘No. Later, later’. I won’t be the first one, but I will be the second or the third.” (Kevin)*.

#### Theme 2: Perceived risk of COVID-19 infection

Most participants consistently depicted COVID-19 as a disease of the frail and elderly. Even if they were to contract COVID-19, most participants expected themselves to recover easily due to their age. Some participants also framed the severity of COVID-19 by juxtaposing it with a common cold. Yasmin said that she would not ‘end up hospitalised’ just from COVID-19, while Hana felt COVID-19 was ‘just like a bad flu’ and that she would be able to ‘get over it’. One participant, Una, talked about the misleading video by the government depicting a COVID-19 infected young woman gasping for air, which she felt misrepresented young adult women’s symptoms of COVID-19.

*“Young, healthy women are not the victims of COVID-19. It’s elderly people who are, they die from it. From my understanding, anyway.” (Una)*.

With the security of low COVID-19 infections in Australia compared to other parts of the world, Gemma found herself ‘desensitised to the fear’ after a long period of the pandemic. Moreover, Flora revealed her COVID-19 vaccine hesitancy concerning her definition of a pandemic:

*“It’s not really a pandemic. A pandemic is where one in five people are dying from it, and there’s not one in five people dying, so I’m not too scared.” (Flora)*.

#### Theme 3: Media’s misrepresentation of COVID-19

The diverse and mixed messaging from the media raised concerns among participants about potential bias, contributing to their hesitancy towards receiving a COVID-19 vaccine. Most participants underlined the media’s manipulative nature and preference for presenting negative news instead of additional vaccine safety information. There was also consensus that news was written in a manner with the sole purpose to draw attention. Jayla, for example, described the media as ‘just looking for ratings’ by ‘doing everything so negative’, a tactic that further increased her mistrust towards the media and hesitance towards the COVID-19 vaccine. The lack of positivity in the way news was presented triggered Yasmin to question the trustworthiness of the media, which she once thought was impartial:

*“It’s really hard to know who to trust anymore… even through COVID I’ve found out how biased media could be, and I didn’t realise that they were even like that, but it has become very clear.” (Yasmin)*.

Many participants also commented that the media’s approach of using ‘hysteria’ and ‘scaremongering’ in the hopes of getting people vaccinated was inappropriate. Varun felt that the media ‘bombarding [COVID-19 vaccine-hesitant persons] with constant fear and hoping that it would create a different response would not work for [him]’. Regardless, most participants were aware of numerous positive vaccination encounters by others but believed the media downplayed these experiences to suit their agendas. Jayla felt the same and commented that the media had not done much to convince her through personal testimonials to be vaccinated. She suggested that a more positive reporting style could encourage her to rethink her hesitancy towards the vaccine.

#### Theme 4: Vaccine-related issues

Most commonly, hesitancy towards the COVID-19 vaccine evolved around specific concerns about the vaccines’ side effect profile and its expected utility. Participants held mixed concerns about the short- and/or long-term safety of the vaccine after being delivered to market so rapidly. As such, participants expressed the need for more time to re-evaluate the safety and effectiveness of the vaccine before receiving vaccination:

*“Short-term effects, I don’t care. Things like that are curable, but if later on down the track, they discovered that it [COVID-19 vaccine] causes something… cancer [or] whatever. Not so much the small effects but big effects… that’s what I’m afraid of.” (Zara)*.

With most participants being women, fertility and women’s health were discussed. Female participants feared the potential effects of the COVID-19 vaccine on their bodies and unborn child/ren. One participant expressed the possibility of guilt should something happen in the future if it related to her receiving a COVID-19 vaccine:

*“I would hate to have something [a COVID-19 vaccine] now and then find out… three or four years down the track that having this vaccine has had some negative effects on [fertility].” (Evie)*.

When asked the question ‘Do you think the COVID-19 vaccine works at all?’, participants appeared bemused and agreed it was a tricky question to answer. Yasmin pointed out that the statistics were not strong enough to support the utility of a COVID-19 vaccine, while Priya explained her rationale as to why the current COVID-19 vaccine was ineffective:

*“From what I’ve heard, you can obviously still get the virus with the vaccination and spread the virus with the vaccination. You just might not know that you have it because the impact of the virus is lessened. So… from my layman’s point of view… I would personally feel like that’s an ineffective vaccine.” (Priya)*.

Many participants adopted a wait and see approach, hoping for additional safety evidence to emerge or for a ‘better’ vaccine. Despite wanting to hold out for as long as possible, most participants revealed that they would be ready to receive a COVID-19 vaccination one day. Varun always felt that ‘there might come a time where [he] must make that decision to [receive a COVID-19 vaccine]’.

#### Theme 5: Inconsistent government messaging and program execution

In Australia, the federal and state governments hold shared and sole responsibilities in national affairs. Consequentially, Australia’s COVID-19 vaccination program is state-run and partially funded by the federal government. Given the complexity of concurrent and exclusive powers held by the governments in healthcare, Nalani recounted how the infamous public disagreement between the Queensland state Chief Health Officer and the Australian Prime Minister on the use of AstraZeneca Vaxzevria COVID-19 vaccine for those under 60 years of age made her more confused. Moreover, Laila stated that when independent peak medical bodies such as ‘the Australian Medical Association discounted what the Prime Minister [comments]’, the inconsistencies and differing statements from multiple levels of governments and non-government expert groups made it difficult for participants to decide on whom to place their trust on.

Despite understanding the need for mass vaccination as a strategy to improve health outcomes and minimise the economic effects from the COVID-19 pandemic, participants noticed that public health messaging was politically charged to work in favour of a higher vaccination rate instead of the concerns of the individual. Jayla described this act as ‘selfish’ and that the governments ‘take and twist the truth’ to achieve a higher vaccination rate. As a result, Varun believed that the issue of blood clots associated with the AstraZeneca Vaxzevria COVID-19 vaccine was downplayed and made less transparent because ‘it interferes with the message that everyone needs to get vaccinated quite quickly’. The government’s ever-changing and sometimes contradictory advice led to much confusion among participants. Yasmin described their advice as ‘wishy-washy’ while Jayla expressed her frustration at the constant change:

*“One day, they say you can [vaccinate with] AstraZeneca. Then the other day, they say ‘Well, you can’t’. And then they say again, because they are falling short of vaccines, they say, ‘Well, hey you know what? You can do it [vaccinate with AstraZeneca]’. I’m not going to be your guinea pig!” (Jayla)*.

#### Theme 6: Social benefits assessment

Despite frequent lockdowns and social distancing, most participants in this study were not adversely affected socially by the restrictions imposed during the pandemic. Relying heavily on social media, Una commented that she felt ‘pretty connected already through the internet… we have Zoom, I’ve got WhatsApp… I’m on video call all the time… so I think my social life probably isn’t going to change with the vaccine or without the vaccine’. Although some participants in pandemic-affected industries were made redundant, many experienced temporary distresses but were able to regain regular employment quickly. With the ability to work from home, many participants considered the COVID-19 vaccine unnecessary for maintaining employment.

Lastly, the lack of immediate benefits following vaccination also made participants hesitant to a COVID-19 vaccination. Many participants commented that their social life would not be affected by receiving or not receiving the COVID-19 vaccine. Quinn remained hesitant because incentives and the removal of restrictions only applied when ‘a significant number’ of the population had received the full course of the COVID-19 vaccine. With restrictions varying between states, some participants including Olive felt that receiving a COVID-19 vaccine ‘earlier than necessary is pointless’ unless restrictions were similar across the country.

### Motivation for the uptake of a Covid-19 vaccine

Motivation towards a COVID-19 vaccine uptake was also explored in the interviews. Participants felt that the provision of tangible benefits post-vaccination, protecting significant others, and the action of vaccine mandates and incentives are major motivators to receiving COVID-19 vaccination.

#### Tangible benefits

The majority of participants expressed the desire to have life return to pre-COVID-19 as soon as possible. Although most participants knew that normality was unachievable immediately after vaccination, Olive mentioned that her meaning of ‘normal’ is having the freedom ‘to go to Sydney on the school holidays’ without having a contingency plan should another snap lockdown occur. A common motivating factor between Varun and Xena was that of immediate removal of lockdown restrictions for those who are fully vaccinated:

*“So, if there’s a law or something that’s concrete and absolute, that… if I get vaccinated or if this amount of people get vaccinated then we won’t have to continuously suffer like this, perhaps I will get it [vaccinated].” (Xena)*.

Another significant motivating factor to be vaccinated against COVID-19 was the ability to travel overseas and interstate. Many participants spoke about how their plans for major international travel were interrupted by the pandemic. Visiting family was also high on participants’ agendas. Yasmin has been unable to visit her sister, who lives interstate, due to state border closures. She adds that ‘if they [state governments] were opening borders up to people who are vaccinated, it might get me a little bit closer to being willing to do it [be vaccinated]’. Participants Kevin and Varun, who are born overseas, also agreed they would willingly receive a COVID-19 vaccine to reunite with family members overseas, especially those who are frail or dying. Varun summarised this motivation best:

*“I think that if I had to do it [get vaccinated] at this point or if I felt compelled to decide for myself that I had to do it, it would be for the ability to see other people… And obviously having the pressure of being apart for so long, it’s hard enough. And some of my family members who are older… that would weigh on my mind. That’s something that could convince me [that] maybe I should get the vaccine.” (Varun)*.

#### Protecting others

Some participants mentioned that protecting close relatives, especially those who are most vulnerable, would be a strong motivator to receive a COVID-19 vaccination. Capri likened this concept to the whooping cough vaccine, where one receives the vaccine to protect one’s own baby or protect the newborns of loved ones. For now, Capri does not have anyone close to her that she feels the need to protect from COVID-19 directly. However, she would ‘do anything [such as being vaccinated against COVID-19]’ if somebody in her family were at a higher risk of severe consequences if they contracted COVID-19.

Despite her hesitancy, Nalani’s motivation towards a COVID-19 vaccination stems from her ‘morality of community-based decisions’. She believed in ‘everyone taking that risk for the betterment of the community’ and that people should ‘get over it’. She went on to elaborate:

*“For me, it’s just literally about… I guess that little old lady down the street that, if I didn’t get it [COVID-19 vaccinated] and I gave it [COVID-19] to her and something [bad] happened, that would be on me. That’s what it comes back to.” (Nalani)*.

#### Mandates and incentives

Many participants agreed that they would receive a COVID-19 vaccination if they had to choose between not being vaccinated and losing employment or being vaccinated and maintaining employment. Most agreed that having an income and associated financial security was more important than resisting a COVID-19 vaccine. Despite being hesitant, Priya was open to receiving a COVID-19 vaccine ‘if it was a prerequisite to work in an industry that [she] wants to work in’.

In addition to mandates, some participants felt that incentives could aid COVID-19 vaccine uptake. Kevin cites an example where financial support payments from the Victorian State Government made residents more willing to be tested for COVID-19 without fear of losing a day’s wage:

*“As a third-party person, you see these things [incentives and support being provided] happen. Okay, the money actually drives people to do [get COVID-19 tested]. So maybe if the government wants to drive people to get vaccinated, money could be the way.” (Kevin)*.

## Discussion

This study aimed to qualitatively explore young adults’ thoughts and feelings about their hesitation to be vaccinated against COVID-19. In particular, the research considers how young adults’ behavioural and social drivers affected their decision-making towards COVID-19 vaccination. This study found that in an information-saturated environment coupled with the unique behaviours of this age group, some young adults in Australia were increasingly challenged when it came to deciding to receive a COVID-19 vaccine during the initial phase of the mass COVID-19 vaccination campaign. This hesitancy was based on the low perceived personal risk of COVID-19 infection, the negative portrayal of COVID-19 vaccines, a distrust of the government’s program planning and messaging, and the perceived limited social benefits from being vaccinated. Our study also allowed participants to describe key enablers and motivators to help improve COVID-19 vaccine uptake within their age group.

### Rethinking COVID-19 vaccine hesitancy in young adults

Throughout this study, our participants have demonstrated their general support for vaccinations. The participants possessed a high level of vaccine literacy and kept up to date with vaccinations for travel and employment. They also considered vaccination as part of the social contract within the society in which they live. Yet, participants remained undecided when specifically receiving a COVID-19 vaccine themselves. Findings in the United Kingdom have presented COVID-19 vaccine-hesitant individuals as disenfranchised members of society [42]. Interestingly, our participants contrasted by being confident, self-aware and possessing a good understanding of the COVID-19 vaccines. This study presents a unique group of COVID-19 vaccine-hesitant individuals aged 25 to 34 years who have perceived themselves to be not heavily impacted by the pandemic. Importantly, their willingness to receive a COVID-19 vaccine has shown to be different to other common vaccines (e.g., influenza, hepatitis B and pertussis).

### Australia’s COVID-19 vaccination program: Perceived flaws

Since the commencement of Australia’s national COVID-19 vaccination program, there has been a prevailing perception among participants that their individual needs have been overshadowed by the government’s overarching objective of attaining a high national vaccination rate. This perception is supported by a substantial body of literature, which underscores a significant lack of transparency regarding vaccine information, including potential side effects and deployment standards. These factors have emerged as crucial determinants of the public’s confidence in their government during the COVID-19 pandemic [33], [43], [44], [45], [46].

Our findings further emphasise the critical role of the media in disseminating clear, accurate, and comprehensive information about the COVID-19 vaccine to the public. Despite the traditionally high trust in the media for delivering truthful public health messages [47], participants predominantly relied on informal channels such as social media, family, and friends for information regarding the COVID-19 vaccine. This reliance mirrors trends observed in parental vaccination behaviours [48]. Given the detrimental role of social media in spreading misinformation about COVID-19 vaccines [49], [50], [51], [52], [53], our findings are novel in that they reveal participants' confidence in their ability to evaluate the intent of media messages critically and to disengage from misleading information if necessary. Nonetheless, these findings underscore the need for governments to incorporate social media as a key platform for disseminating crisis communications and fostering public trust [54]. Furthermore, our research confirms that a synergistic collaboration between governmental bodies and media agencies could enhance the effectiveness of communication strategies in future public health initiatives, including the rollout of new vaccines. Such a collaborative approach would ensure that public health messages are accurate, reliable, and resonate with and comprehensively address the community's concerns.

### Vaccine-related issues

Our findings have demonstrated that pivotal vaccine-related negative events (such as blood clots from AstraZeneca Vaxzevria) have deeply ingrained further doubts and mistrust in the safety aspect of all COVID-19 vaccine brands in Australia. Despite Europe and Australia adopting an age-dependent vaccine allocation policy as a solution to blood clotting issues, vaccine hesitancy continued to persist [55], [56], [57]. Such observations suggest that countries and their governments must be quick to react to the expressed sentiments of their citizens and come up with fair and equitable access to safe vaccines for everyone. Further, female participants continued to be concerned about the COVID-19 vaccine’s effects on women’s health and fertility despite robust safety data in childbearing women [58], [59], [60].

We also noted that participants were open to receiving a COVID-19 vaccine in the near future but, in the interim, opted to ride on the benefits of plausible immunity afforded by early adopters of the vaccine observed in other literature [61]. Participants displayed awareness of the social responsibility of becoming vaccinated and being an example for others. Similar to other studies, our participants were fully aware of the contradiction of their thoughts and inconsistencies of their actions [38], [62], [63]. Confirming findings in other recent literature [64], ‘Vaccine Delayers’ seems to be a more precise construct for our study’s participants, as they delay vaccination until a significant benefit presents itself.

### The quest for more information… and bias

As observed in this study, the abundance of conflicting messages coupled with participants’ proactive search for further evidence is an important factor feeding vaccine hesitancy, resulting in a cognitive bias known as omission bias, whereby individuals put disparate weight on some claims but not others [65], [66]. When Hana felt that receiving a COVID-19 vaccine was more dangerous to her health than smoking, this antagonistic feeling was also observed among parental vaccine-hesitant mothers and young adults towards the seasonal influenza vaccine [67], [68]. Given our findings, future research around COVID-19 and other public health messages should aim to standardise the information content across all media platforms to reduce such further practice of omission bias by the public.

### Recommendations to increase COVID-19 vaccination among young Australian adults

The current study found that COVID-19 vaccine-hesitant participants were motivated by a range of internal, external and structural factors. However, the most powerful motivator was identified to be external influence.

First, the provision of tangible personal benefits (i.e., reinstatement of interstate/overseas travel) immediately upon vaccination was a central motivating factor. While other literature identified the importance of the collective benefits of vaccinations [69], our participants’ willingness to forgo previously mentioned barriers to a COVID-19 vaccination once a suitable personal benefit presents itself highlighted how this collective action becomes less persuasive in the context of receiving a COVID-19 vaccination. Recent studies demonstrated an association between personal gains and a higher COVID-19 vaccine uptake [70], [71]. This behaviour, known as ‘utilisation delay’ [72], was observed when the reopening of international travel saw an uptick in COVID-19 vaccination uptake [64], [73]. As participants are reluctant but persuadable with the right incentives, pro-vaccination communication skewed towards personal benefit [71], [74], [75] could be a particularly effective persuasion tool compared to a directive to receive a COVID-19 vaccination. This points to the need to consider age-specific variable motivations when creating incentives to promote COVID-19 vaccination uptake across the Australian population.

Like other studies [76], [77], our findings also identified the external role and value of personal COVID-19 vaccination experiences as a determining factor in COVID-19 vaccine acceptance. Participants reveal how video testimonials of other young adults sharing their intentions to have the COVID-19 vaccine would be a more effective form of persuasion. As such, public health messaging could utilise influential COVID-19 vaccine ambassadors to engage with these young adult populations through personalised vetted social media engagements, as seen in a recent study [36]. Interestingly, participants also provided useful strategies such as using live debates between vaccine manufacturers and the public, easy-to-understand infographics and promoting the COVID-19 vaccine through positive emotional appeals.

The desire for increased liberty of movement suggests that structural motivators such as vaccine mandates can effectively motivate young adults. Such structural incentives have shown to be effective when used with tangible personal benefits such as interstate/overseas travel and accessing preferred employment [62]. While vaccine mandates can influence COVID-19 vaccine uptake [78], our findings demonstrate that these mandates are only effective when in line with participants’ personal goals and interests.

### Implications for policy and practice

The findings of this study demonstrate the diversity of reasons young adults in Australia are hesitant towards the COVID-19 vaccine. Exploring the decision-making behaviours of young adults towards a COVID-19 vaccination can create a new evidence-based social contract to encourage vaccine acceptance [79]. Effectively harnessing the lessons learned from Australia’s initial rollout of its mass COVID-19 vaccination program through strategies grounded on transparency and trust and the use of participatory and engagement activities will be central to bridging the gap between vaccine hesitancy and vaccine acceptance. Using Australia’s initial hesitance to the COVID-19 vaccine as a case study, it would be prudent for governments to address such challenges and begin strategising solutions as part of the country’s public health engagements in pandemic and health crisis emergency response.

### Summary

To date, a prominent strategy for addressing vaccine hesitancy involves a thorough exploration of the cognitive and affective dimensions of individuals' attitudes towards vaccines and their personal beliefs on this matter. In the context of addressing COVID-19 vaccine hesitancy among young adults, it is recommended to employ tailored communication tools that cater to the specific factors contributing to this hesitancy. Additionally, the utilisation of innovative communication methods is advised to formulate a comprehensive communication strategy [80]. In line with the findings of this study, individuals exhibiting hesitancy towards the COVID-19 vaccine may be classified as being initially reluctant but potentially amenable to persuasion, particularly if they are presented with highly targeted and personalised incentives for personal benefit. It has been suggested that persuasive communication when tailored to emphasise individual benefits, may prove effective compared to communication framed as a cue to action. Such personalised messaging strategies are recommended for segments of the population that continue demonstrating significant hesitancy towards the COVID-19 vaccine [71], [74].

Over time, once initial fears of the safety and efficacy of the COVID-19 vaccines (new adverse events notwithstanding) start to dissipate gradually; there will inherently be an increase in willingness to accept vaccination as it becomes an accepted normative action towards the fight against COVID-19 [81], [82]. The findings from this study can provide public health professionals and governments with the knowledge to design suitable policies and create favourable motivation options for improved vaccine uptake within this unique group of young adults in Australia.

### Limitations

Due to the voluntary nature of this research, there is a potential for self-selection bias. In addition, with a majority female participant pool, it would be ideal to have more male participants to offer additional insights into their hesitancy towards the COVID-19 vaccine. It is noted that our study’s participants resided in three Australian states at a sampling time period where varying levels of COVID-19 infections and social restrictions were present. Further, most parts of this study took place when the availability of the Pfizer Comirnaty COVID-19 vaccine was in limited supply, and not all participants were yet eligible to receive a vaccination. Nevertheless, the enablers and barriers to vaccine acceptance identified in this study should be adequate to form a basis for subsequent studies on vaccine implementation research (i.e., the COVID-19 vaccine booster program, annual influenza vaccination and the varicella-zoster vaccine uptake in elderly populations). They can be particularly useful in countries with inconsistent or inequitable COVID-19 and other vaccine resources.

## Conclusion

We found that young adults see the importance of the COVID-19 vaccine as a means to returning to pre-pandemic livelihoods but, at the same time, question their willingness to accept a COVID-19 vaccine for themselves. This research demonstrates the dilemma young adults face while being aware of the societal benefits of COVID-19 vaccination and the inconsistencies of their actions. Although willing and not resistant to the principles of the COVID-19 vaccine social contract, young adults were found to set aside this common good until such a time as vaccination was more urgently required. This research also demonstrates the importance of examining the enablers and barriers of COVID-19 vaccine uptake to fully address the complexities of vaccination decision-making. Despite the slow start, considerable success in achieving a high COVID-19 vaccination rate in Australia has been achieved, and it is hoped that the findings of this study uncover the unique challenges surrounding vaccine hesitancy and health decision-making behaviours in adults across the age spectrum. Understanding and reacting to real-time public health challenges provides important transferrable lessons that can be used to inform and strengthen evidence around best practices in future health programs where compliance to medicines or health activity uptake *en masse* is critical to operational success. The unpredictable threat of emerging public health challenges calls for all cross-cutting health program activities to be conducted seamlessly between authorities, health media communicators and pharmaceutical companies. Global health policy leaders must learn from these challenges and refine and continually review existing systems (e.g., incorporating age-specific strategies and favourable motivations) as part of their readiness and response plan to future shocks and emerging public health challenges.

## Funding

This research received no external funding.

## Declaration of Generative AI and AI-assisted technologies in the writing process

During the preparation of this work the author(s) used Grammarly in order to correct grammar and punctuation for clear readability of this manuscript. After using this tool/service, the author(s) reviewed and edited the content as needed and take(s) full responsibility for the content of the publication.

## CRediT authorship contribution statement

**Zachary Sum:** Conceptualization, Methodology, Formal analysis, Investigation, Data curation, Writing – original draft, Writing – review & editing, Visualization, Project administration. **Ernesta Sofija:** Conceptualization, Methodology, Formal analysis, Writing – review & editing, Visualization, Project administration. **Bernadette Sebar:** Conceptualization, Methodology, Formal analysis, Writing – review & editing, Visualization, Project administration.

## Declaration of competing interest

The authors declare that they have no known competing financial interests or personal relationships that could have appeared to influence the work reported in this paper.

## Data Availability

Data will be made available on request.
